# The Regenerative Effect of Various Barrier Membranes With and Without Bone Grafting in Critical Size Defects in Rabbit Calvaria

**DOI:** 10.1002/cre2.70306

**Published:** 2026-02-26

**Authors:** Abdelsalam Elaskary, Rola Al Habashneh, Tasneem Sharairi, Moataz Meabed, Mahmoud Akram Khodir, Ahmed Zaki, Mohammed Alorjani, Bassem Elfahl, Malek Hudieb, Alhassan Diab, Mihad Ibrahim

**Affiliations:** ^1^ Private Practice Alexandria Egypt; ^2^ Department of Periodontology Jordan University of Science & Technology Irbid Jordan; ^3^ Department of Oral and Maxillofacial Surgery Alexandria University Alexandria Egypt; ^4^ Centre of Excellence for Drug Preclinical Studies (CE‐DPS), Pharmaceutical and Fermentation Industry Development Centre City of Scientific Research and Technological Applications New Borg El Arab Egypt; ^5^ Department of Histopathology Jordan University of Science & Technology Irbid Jordan; ^6^ Department of Periodontology Tanta University Tanta Egypt; ^7^ Department of Preventive Dentistry Jordan University of Science &Technology Irbid Jordan; ^8^ Department of Periodontics and Oral Medicine British University in Cairo Cairo Egypt; ^9^ Department of Oral Medicine and Periodontology, Faculty of Dentistry Cairo University Cairo Egypt

**Keywords:** bone regeneration, bone substitute, cortical membrane, experimental animal model, guided bone regeneration, histology

## Abstract

**Objectives:**

This multicentre randomized controlled clinical trial aims to investigate the regenerative effects of various thicknesses and types of barrier materials with and without bone grafting in a rabbit calvaria model.

**Materials and Methods:**

One hundred male rabbits were partitioned into two groups: one without bone graft (NB) and one with bone grafting (BG). The groups were further divided into five subgroups, *n* = 10 each: C (control); SC (0.3 mm single‐layered collagen); DC (0.6 mm double‐layered collagen); L1 (0.5 mm cortical collagenated bone barrier); and L2 (1.0 mm cortical collagenated bone barrier). In all experimental groups, each distinct type of barrier was applied following the creation of a 10 mm circular defect in the calvaria of each rabbit. After 24 weeks, the calvariae were examined by histologic and histomorphometric analyses.

**Results:**

The utilization of cortical bone barriers increased bone formation in all experimental groups. For Group NB, the histological score significantly differed among subgroups (*p* < 0.001). L1 and L2 subgroups had more favorable histological scores than the control groups (*p* < 0.001). Furthermore, the L2 subgroup had a higher histological score than the SC subgroup (*p* < 0.001). In Group BG, histological score significantly differed among subgroups (*p* < 0.001). DC, L1, and L2 subgroups had higher histological scores than the controls (*p* < 0.02), (*p* < 0.001), and (*p* < 0.001), respectively. The L2 subgroup had a higher histological score than the SC subgroup (*p* < 0.01). The BG group had significantly higher histological scores overall compared to the NB group based on barriers (*p* < 0.05).

**Conclusions:**

Within the limits of this model, the 1.0 mm cortical lamina barrier demonstrated the most favorable regenerative performance, consistently achieving higher histologic scores and more advanced tissue maturation than thinner cortical lamina or collagen membranes. These findings indicate that barrier architecture, particularly thickness and mechanical stability, plays an important role in promoting predictable bone regeneration.

**Abbreviations**
ARRIVE: animal research: reporting of in vivo experiments
BG: bone graft group
C: control
DC: double collagen
H&E: hematoxylin and eosin
IV: intravenous
L1: cortical lamina barrier 0.5 mm thickness
L2: cortical lamina barrier 1 mm thickness
NB: no bone graft group
PASS: primary closure, angiogenesis, space maintenance, stability
PTFE: polytetrafluoroethylene
SC: single collagen
VST: vestibular socket therapy

## 1 Background

The rehabilitation of partially or completely edentulous patients with implant‐supported prostheses is now a well‐established standard of care, demonstrating predictable and favorable long‐term outcomes (Chiapasco and Casentini 2018). However, achieving optimal functional and esthetic results requires precise implant placement, which depends on addressing deficiencies in bone volume and keratinized mucosa (Tavelli 2023). A key principle of periodontal regenerative methodologies is the establishment and maintenance of a well‐isolated environment during wound healing (Wang and Boyapati 2006). Barrier membranes have therefore become integral to regenerative procedures, functioning to exclude rapidly proliferating epithelial and connective tissue cells while preserving space for osteogenic repopulation (Fraser et al. 2022). Barrier stabilization is essential in guided bone regeneration (GBR) (Li et al. 2018) to prevent micromovement and ensure selective cell migration while excluding fibroblasts, thereby minimizing fibrous tissue encapsulation.

The foundational principles of successful GBR (Schultz 1993) have been extensively studied using a canine supra‐alveolar periodontal defect model, in which specific defects were created to observe spontaneous tissue regeneration (Wikesjö et al. 1998). Non‐resorbable polytetrafluoroethylene (PTFE) membranes, with or without titanium reinforcement, were introduced to provide adequate space maintenance and prevent collapse (Nyman et al. 1982). Despite yielding acceptable results, they require a second surgical procedure for removal, which increases patient morbidity and has been linked to poor soft tissue responses (Chiapasco et al. 1999).

Resorbable collagen membranes were subsequently developed as an alternative and are characterized by excellent biocompatibility and favorable tissue integration (Bunyaratavej and Wang 2001). However, their relatively rapid biodegradation limits their ability to maintain regenerative space in larger defects for an adequate time. To overcome these limitations, double‐layered collagen membranes have been proposed to increase structural resilience and prolong barrier function (Buser et al. 2004). Despite demonstrating improved performance, double‐layered membranes still show limitations in space maintenance when used in defects requiring substantial bone augmentation (Sbricoli et al. 2020)

Barrier membranes derived from xenogeneic sources are characterized by resistance to osteoclastic activity, the ability to remodel, and functionality until bone maturation is achieved (Hallman et al. 2002). More recently, cortical xenograft barriers derived from porcine or equine sources have been introduced, offering a combination of biological compatibility and mechanical rigidity advantageous for GBR applications (Schuh et al. 2021). Through proprietary processing methods that prevent hydroxyapatite crystal ceramization, these xenogenic cortical barriers maintain structural integrity while undergoing slow, controlled resorption (Korner et al. 2022; Rossi et al. 2023; Schuh et al. 2021). Their rigidity and slow degradation support clot protection and space maintenance throughout early healing, while their resorbable nature eliminates the need for re‐entry surgery, thus reducing patient morbidity (Rossi et al. 2023). These benefits have been clinically and radiographically demonstrated in several clinical trials during immediate implant placement (Elaskary et al. 2021).

Critical‐size defects are defined as the smallest osseous wounds that cannot heal spontaneously within a specific timeframe or the animal's lifetime. These defects are frequently created in animal models to evaluate the effectiveness of various GBR techniques on bone healing (Schmitz and Hollinger 1986; Spicer et al. 2012)

Previous studies have investigated multiple regenerative strategies in critical‐size defects, including bone grafting with or without collagen membranes (Bosco et al. 2016; Gholami et al. 2021) and the use of collagen membranes with or without bone substitutes (Botticelli 2022; Udeabor et al. 2020). However, despite their increasing clinical use, cortical xenograft laminae have not been systematically tested in standardized rabbit calvarial critical‐size defects, particularly with respect to barrier thickness and the adjunctive use of bone grafts. Despite the growing adoption of cortical xenograft barriers in regenerative procedures, the current literature lacks high‐quality, comparative in vivo studies assessing their performance under standardized conditions, particularly in critical‐size defects (Retzepi and Donos 2010; Roca‐Millan et al. 2020; Zitzmann et al. 2003)

Addressing this gap is crucial for optimizing clinical decision‐making in regenerative implant therapy. Therefore, this in vivo study aimed to histologically evaluate and compare the regenerative effects of collagen and cortical xenograft barriers of varying thicknesses, with and without bone grafting. It was hypothesized that cortical xenograft barriers of 0.5 mm and 1 mm thickness, combined with bone grafting, would result in greater bone regeneration in critical‐size defects compared to single or double‐layered collagen membrane with and without bone grafting.

These findings could aid in the selection of barrier types and thicknesses by practitioners, especially in cases requiring ridge augmentation and GBR. Furthermore, the results may provide a foundation for future clinical trials evaluating membrane type and thickness in the management of alveolar defects.

## 2 Materials and Methods

A total of 100 healthy male, 6‐month‐old New Zealand white rabbits weighing approximately 3.0 kg were utilized in this study. Male rabbits were selected to avoid potential hormonal fluctuations in females that could affect inflammatory responses and bone healing. Fifty rabbits were obtained from the Alexandria Borg El‐Arab University Veterinary Department, and 50 from the Research Center and Techno University in Irbid, Jordan. A multicenter design was adopted to enhance the generalizability of findings and minimize center‐specific biases in surgical technique, animal handling, and histologic processing. The animals were housed in individual cages at a controlled temperature of 25°C and kept on a standard soft diet with access to water ad libitum. All experimental procedures were approved by the Jordan University of Science and Technology Animal Care and Use Committee (JUST‐ACUC‐192) and the Institutional Animal Care and Use Committee of the Alexandria Borg El‐Arab University Research Center (67‐4J‐9022). The study followed the Animal Research: Reporting of In Vivo Experiments (ARRIVE) (Delgado‐Ruiz, Calvo‐Guirado, & Romanos) guidelines to ensure transparency and reproducibility. Clinical trial registration was not applicable for this experimental animal study.

### 2.1 Experimental Design and Centers

This multicenter randomized controlled in vivo study was conducted at the animal facilities of Jordan University of Science and Technology and the Alexandria Borg El‐Arab University Veterinary Department. All histologic and histopathologic procedures were performed in the histology laboratory of the Faculty of Medicine and the Alexandria Borg El‐Arab University Research Center.

The biomaterials used included a cortical bone barrier with 0.5 mm thickness (L1; OsteoBiol Lamina Soft, Tecnoss, Giaveno, Italy), a cortical bone barrier with 1.0 mm thickness (L2; OsteoBiol Lamina Curved, Tecnoss), and a resorbable collagen membrane with 0.3 mm thickness used either as a single layer or in double thickness of 0.6 mm (DC) (Mem‐Lok, BioHorizons, USA). The bone graft material was a xenogeneic particulate graft (MinerOss X, BioHorizons, Birmingham, AL, USA).

### 2.2 Sample Size Calculation

The minimum sample size was calculated based on a previous study (Kamal et al. 2019) evaluating bone healing in rabbit calvarial defects using various bone substitute materials compared to a negative control. Based on histologic new bone formation values of 4.3 ± 6.4% in the control group and 21.0 ± 11.9% in the particulate bone graft group, and adopting a power of 80% *β* = 0.20 with a significance level of 5% *α* = 0.05, the minimum required sample size per subgroup was calculated to be seven. This was increased to 10 per group to enhance statistical power and account for possible loss, yielding a total of 50 rabbits, divided into 10 subgroups, 5 per main group G*Power, Version 3.1.9.6.

### 2.3 Inclusion and Exclusion Criteria

Inclusion criteria required that all animals be free from systemic diseases, congenital abnormalities, or any signs of infection before surgical intervention. Only rabbits with fully developed calvarial bone structures were selected to ensure model consistency. Exclusion criteria included pre‐existing health conditions, abnormal behavior, or physiological instability before surgery, as well as the development of severe postoperative complications such as persistent infection, wound dehiscence, neurological symptoms, or respiratory distress that could interfere with healing assessments. These animals were humanely euthanized and excluded from both qualitative and quantitative histomorphometric analyses. Only animals that completed the 6‐month follow‐up were included in the final data set.

#### 2.3.1 Randomization

Allocation of the treatment modalities was randomized according to a computer‐generated sequence (www.randomization.com). Rabbits were allocated into two main groups: Group 1 (NB), in which no bone grafting was performed, and Group 2 (BG), in which grafting was applied. Within each main group, animals were further assigned to one of five membrane conditions (C, SC, DC, L1, L2). The housing location of the rabbits was also randomized within the facilities to minimize environmental confounding factors such as light exposure, noise, and caretaker variability. All animals received identical diets, housing conditions, and postoperative care protocols.

### 2.4 Blinding

Group allocation was managed by a researcher (MI) not involved in the surgical procedures or outcome assessments. The histologic scoring and image analysis were performed independently by a single, blinded histopathologist (MA). Data analysis was conducted using coded datasets, and group identities were revealed only after all statistical analyses were completed.

### 2.5 Surgical Procedures

All surgical procedures were conducted under general anesthesia and aseptic conditions. Rabbits were anesthetized via intramuscular injection of ketamine (Ketalar, Pfizer, UK) at 35 mg/kg and xylazine (Xylazine 2%, Alfasan, Woerden, Holland) at 5 mg/kg of body weight. Once general anesthesia was established, an electric shaver was used to shave the rabbit's head. The skin was disinfected with a povidone iodine solution (HiGeen Ltd. Company, Hungary). A 5 cm midline incision was made along the cranial vault using a 15 C surgical blade, and a cutaneous flap was elevated. The periosteum was carefully incised and reflected bilaterally with a small periosteal elevator to expose the calvarial bone.

A circular critical‐size defect, 10 mm in diameter, was created in the parietal bone of each rabbit using an 11 mm stainless‐steel trephine drill (Neodent) attached to a low‐speed micromotor (speed: 850 rpm; torque: 35 Ncm; NSK Surgic AP). Continuous saline irrigation was used during drilling to prevent overheating. The defect was prepared to full calvarial thickness (approximately 1.7–2.0 mm), and drilling was continued until the dura mater was just exposed, ensuring a standardized full‐thickness (bicortical) critical‐size defect in each animal. Thus, defect depth was standardized relative to the local bone thickness rather than a fixed millimetric depth, avoiding partial‐thickness variability among specimens. Special care was taken not to injure the underlying dura mater. A single unilateral defect was created per animal to prevent cross‐contamination and to ensure independent data points.

In the BG group, the xenogeneic bone graft material was placed into the surgically created defect before barrier placement. The type of barrier assigned to each animal was determined by the randomization protocol. All membranes were stabilized using 3‐mm titanium fixation pins (Auto‐tacs, BioHorizons, Birmingham, AL, USA), with the same pin type and length used for all groups. Despite minor differences in membrane thickness (0.3–1.0 mm), fixation was achieved without difficulty, and all membranes exhibited stable adaptation without dura penetration. Two fixation pins were used routinely, one on each side of the defect. When full adaptation was not achieved with two pins, a third pin was placed. This was required in seven specimens (7.8% of all analyzed defects), four in the NB‐L2 subgroup and three in the BG‐L2 subgroup. No additional pins were required in the SC, DC, or L1 groups. All membranes were trimmed with sterile scissors using a calibrated sterile template to standardize their dimensions across groups. Each cortical lamina (L1 and L2) was contoured to the curvature of the calvarium to ensure close adaptation to the physiologic bony envelope and to avoid augmentation beyond it.

**Figure 1:**
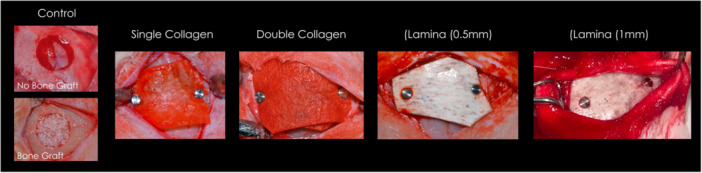
Clinical photographs demonstrating a representative overview of the surgical procedure for each experimental group following membrane placement and prior to flap closure.

In the control group (C), no membrane was placed over the defect. Representative intraoperative images for each group are presented in Figure 1.

Following the barrier placement, the flap was sutured using 4‐0 silk sutures (Atramat, Mexico). All rabbits were housed at room temperature and monitored until stable during the immediate post‐operative period. Once stable, they were transferred to their respective cages and provided with ad libitum access to dry food and water. Following surgery, all animals received postoperative care to ensure pain management and prevent infection. Enrofloxacin 10 mg/kg, subcutaneously, was administered once daily for 5 days as an antibiotic prophylaxis. Analgesia was provided using meloxicam 0.2 mg/kg, subcutaneously once daily for 3 days to minimize discomfort.

The euthanasia process was performed according to the American Veterinary Medical Association guidelines(Kirkwood 2013). Premedication was administered using ketamine 65 mg/kg and xylazine 4 mg/kg subcutaneously in the neck region to ensure deep sedation and minimize distress. Once unconsciousness was confirmed, euthanasia was performed via intravenous administration of pentobarbital sodium at a dose of 120 mg/kg (Streuli Pharma AG, Uznach, Switzerland) (Kirkwood 2013). Following euthanasia, block resections of the skulls were performed using a surgical bur attached to a low‐speed electric handpiece.

### 2.6 Histologic Processing

**Figure 2:**
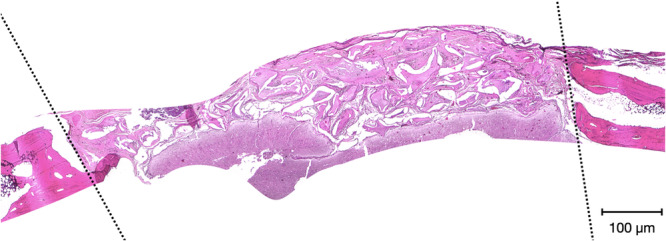
Representative photomicrograph of a histological sample harvested at the 6‐month time point from the L2 group (1.0‐mm cortical lamina), stained with hematoxylin and eosin. Dotted lines indicate the boundaries of the region of interest evaluated by the histopathologist.

Each block biopsy, including the bone defect with the membrane, was decalcified in bone decalcification solution (Diapath S.p.a., Martinengo, Italy) for 14 days. After routine processing, each calvarial block was oriented so that the line connecting the fixation pin tracts defined the mediolateral axis of the defect. Slices were obtained from the central part of each healed bone defect using a saw (Exakt saw 312, Exakt Apparatebau GmbH, Norderstedt, Germany), embedded in paraffin, sectioned longitudinally into multiple 5‐μm‐thick sections, and stained with Hematoxylin and Eosin. For qualitative analysis of the bone regenerative process, tissues were examined by light microscopy (Optika, B‐150DB, Italy), and the entire section was evaluated. Photomicrographs of the tissue sections were captured at 4× magnification. The slides were evaluated both qualitatively and quantitatively for overall histomorphometric outcomes, as shown in Figure 2, while software was used to calculate semi‐quantitative measurements of newly formed bone (ImageJ, Maryland, USA).

Peripheral areas were not evaluated because their revascularization and ossification kinetics differ from those of the avascular central core. The following histologic characteristics were assessed: preservation of compact bone tissue; continuity of the interface between soft tissue, biomaterial elements (residual membrane or graft particles, depending on the group), and newly formed bone trabeculae; presence of osteocytes in lacunae; osteoblastic activity; presence of young blood vessels and fibrocytes; maturity of newly formed connective tissue; and degree of defect filling with new bone.

The border criterion was evaluated as a single composite score (0–2) reflecting the overall organization and integration among these components, where higher scores indicated a well‐defined, continuous, and mature interface between bone, biomaterial, and connective tissue. The histologic characteristics were scored according to criteria originally described by Zoran et al. (2014) for semiquantitative analysis. This scoring system, although initially developed for GBR, has been applied in similar studies and is considered appropriate for assessing histologic outcomes in this model.

For quantitative histomorphometric analysis, the total defect area was delineated as a region of interest (ROI) using the Polygon Selection tool in ImageJ. The ROI corresponded to the original circular surgical defect, identified by the preserved trephine margins in the calvarial bone. Measurements were consistently taken from the central section of each defect, as described in the section on sectioning standardization, to ensure comparable depth and to avoid peripheral variability in revascularization and ossification. To account for inter‐animal differences in calvarial thickness, all histomorphometric outcomes were expressed as area percentages relative to the total defect area, allowing direct comparison among groups independent of individual bone thickness.

New bone was defined on Hematoxin and Eosin‐stained sections as eosinophilic mineralized matrix containing osteocytes within lacunae and, where present, lined by osteoblasts. Unmineralized osteoid seams without osteocytes, fibrous connective tissue, residual graft particles, and membrane remnants were excluded from the measurements.

**Percentage Bone Fill (%BF):** This area‐based parameter represents the ratio of newly formed bone area to the total defect area within the ROI and reflects the extent of bone regeneration inside the defect. It was calculated as:

%BF=[NewBoneArea/TotalDefectArea]*100

Defect Closure (%DC): Linear closure was assessed by measuring the shortest distance between the newly formed bone fronts across the central diameter of the defect using the Straight Line tool in ImageJ. The result was expressed as a percentage of the original defect diameter:

%DC=[1−LinearGapWidth/10]*100

All measurements were performed in triplicate by the same blinded examiner. The mean values from three consecutive central sections were used for each specimen to minimize sampling error. This ensured consistent analysis across groups and improved reproducibility of the histomorphometric evaluation.

The histologic scoring was performed by the same histopathologist (MA). To ensure reproducibility, a standardized protocol with clear criteria and representative images for each score was used. The examiner was trained and calibrated using 10 representative slides before analysis. For each criterion, a score ranging from 0 to 2 was assigned, yielding a maximum score of 14 points. The final histologic score was expressed as a percentage of this maximum value by summing the categorical scores and dividing by 14. The scoring criteria are summarized in Table 1.

**Table 1:** The criteria for the estimation of histologic characteristics.

Histologic property	“0”	“1”	“2”
The compactness of bone tissue	Not preserved	Partially preserved	Preserved
The borderline between soft tissue, elements^a^, and newly formed bone	Unclear	Partially clear	Clear
Presence of osteocytes in lacunae	Empty	Partially complete	Complete
Osteoblastic reaction	Absent	Moderate	Marked
Presence of young blood vessels, fibroblasts, fibrocytes	Not formed	Partially marked	Marked
Maturity of newly formed connective tissue	Young, immature	Partially marked	Mature
Defect filling with newly formed bone tissue	Defect filling = 1/3	Defect filling < 2/3	Defect filling > 2/3

^a^ Elements: Biomaterial remnants present within the defect are either residual membrane fragments or graft particles or both depending on the experimental subgroup.

### 2.7 Statistical Analysis

Data were analyzed using SPSS software (IBMCorp Ibm 2017). The Shapiro–Wilk test (Shapiro and Wilk 1965) was applied to assess the normality of quantitative variables. Because all variables demonstrated non‐normal distributions, non‐parametric statistical tests were used (Field 2024).

Descriptive statistics are presented as minimum, maximum, median, and interquartile range (25th–75th percentiles). For comparisons between the two main groups (NB vs. BG), the Mann–Whitney test (Mann and Whitney 1947) was applied. For comparisons among the five subgroups within each main group (C, SC, DC, L1, L2), the Kruskal–Wallis test (Kruskal and Wallis 1952) was used. When significant differences were detected, post‐hoc pairwise comparisons were performed using the Dunn–Šidák test with Šidák‐adjusted *p*‐values to control for multiple comparisons(Dunn 1964).

An alpha level of 0.05 was used to determine statistical significance. During sample size calculation, a beta error of 20% (power = 80%) and a significance level of 5% were assumed (Curran‐Everett 2020).

## 3 Results

### 3.1 Animal Observations

**Figure 3:**
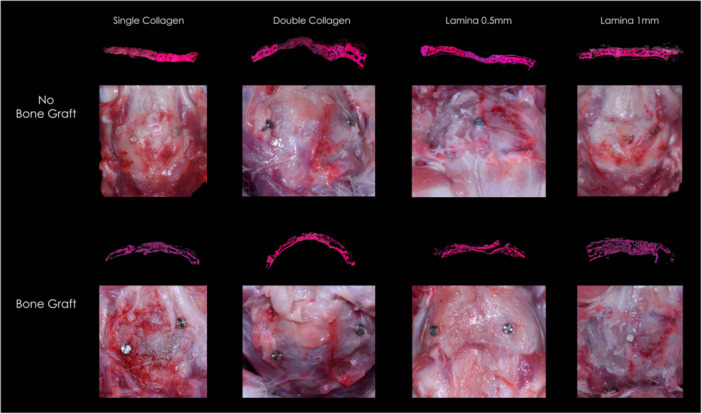
Clinical photographs and corresponding photomicrographs of all membrane groups at the 6‐month time point. Histological sections were stained with hematoxin and eosin, and representative fields were merged into a single composite image for each group.

Of the initial 100 animals, 90 completed the 6‐month follow‐up (48 NB, 42 BG). Ten rabbits (10%) were excluded due to postoperative complications. In the BG group, 8 exclusions were infection‐related (1 C, 1 SC, 2 L1, 4 L2). The remaining animals showed uneventful postoperative healing with no clinical signs of infection or morbidity. Representative images at the 6‐month endpoint are provided in Figure 3.

### 3.2 Post‐Surgical Complications

Six rabbits were euthanized within 1–2 days post‐surgery due to neurological or respiratory complications. Two animals were euthanized because of severe postoperative infection, and two were excluded due to wound dehiscence that prevented reliable sample collection.

### 3.3 Histologic Analyses

**Figure 4:**
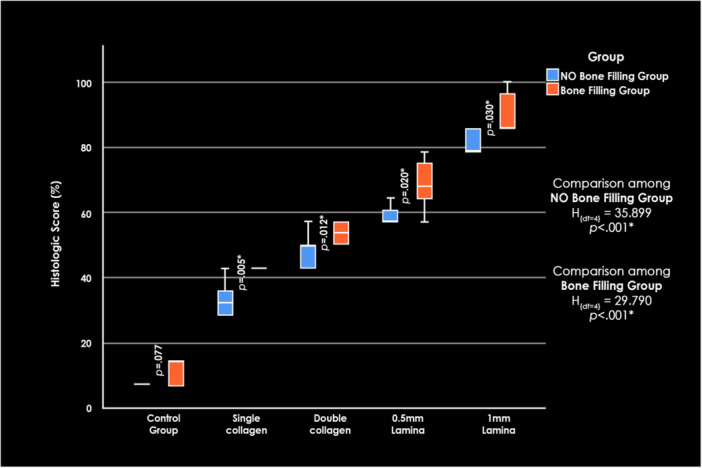
Histological score percentage comparisons among all experimental groups. Box‐and‐whisker plot illustrating the distribution of histological score percentages across the studied subgroups.

Figure 4 demonstrates overall comparisons between the control and experimental groups.

### 3.4 The NB Group (*N* = 48)

Histologic assessment revealed the presence of residual membrane in the SC group. An island of mature new bone featuring scattered osteoblasts was observed within the surgical defect area. At the periphery of the defect, newly formed bone was continuous with the adjacent pristine bone. No osteoclasts or areas of significant bone resorption were noted. Membrane fragments were also observed in the DC group, where new bone formation surrounded the membrane remnants predominantly at the defect periphery, with partial continuity to the native bone.

In the L1 subgroup, approximately 9 mm of the cortical bone barrier remained, appearing discontinuous. Mature bone islands were present centrally, with new bone extension toward the defect margins. Scattered osteoclasts were detected adjacent to cortical remnants. The L2 subgroup similarly exhibited partially resorbed cortical lamina with evident new bone formation at both defect edges and clear continuity with adjacent pristine bone.

Histologic scores increased progressively across subgroups, with the highest values in the L2 group and the lowest in the control group. The Kruskal–Wallis test confirmed significant differences among subgroups (*p* < 0.001).

### 3.5 Quantitative Histomorphometry

Median bone fill and defect closure percentages increased consistently from the control subgroup through SC, DC, L1, and L2. The L2 group demonstrated the highest median bone fill, 68.7% (IQR 67.5%–69.8%), and defect closure 64.1% (IQR 63.2%–64.5%). All comparisons showed statistically significant differences across subgroups (*p* < 0.001). Detailed values are presented in Tables 2 and 3.

**Table 2:** Quantitative histomorphometric analysis.

Fill (%)	Group					Test of significance *p*‐value
	Control (a)	Single collagen (b)	Double collagen (c)	0.5 mm lamina (d)	1 mm lamina (e)	
**No bone graft group**						
– *n*	10	10	8	10	10	
– Min–max	11.50–13.00	30.80–35.00	45.40–48.00	52.80–56.20	66.90–70.10	*H*_(df=4)_ = 45.102
– Median	12.15^a,b^	32.90^a,b,c^	46.90^b,c,d^	54.90^c,d,e^	68.70^d,e^	*p* < 0.001*
– 25th –75th percentile	11.90–12.60	31.90–33.80	46.05–47.35	53.70–55.60	67.50–69.80	
**Bone graft group**						
– *n*	9	9	10	8	6	
– Min–max	18.40–20.40	45.70–49.50	60.20–63.20	71.80–76.10	87.20–90.70	*H*_(df=4)_ = 39.261
– Median	19.20^a,b^	47.30^a,b,c^	61.65^b,c,d,e^	74.20^c,d,e^	89.45^c,d,e^	*p* < 0.001*
– 25th –75th percentile	18.70–19.80	46.80–48.50	60.80–62.40	72.90–75.40	88.20–90.30	
Test of significance	*Z*_(MW)_ = 3.674	*Z*_(MW)_ = 3.674	*Z*_(MW)_ = 3.554	*Z*_(MW)_ = 3.554	*Z*_(MW)_ = 3.254	
*p*‐value	*p* < 0.001*	*p* < 0.001*	*p* < 0.001*	*p* < 0.001*	*p* < 0.001*	

*Note: n*, Number of patients. Min–max, Minimum–maximum. *Z*_(MW)_ = *Z* of the Mann–Whitney *U* test. H = Kruskal–Wallis H test. Pairwise comparisons: Superscript alphabets (a, b, … etc.) are assigned to groups in sequence: (a) for the control group, (b) for the single collagen group, and (c) for the double collagen group, (d) for the 0.6 mm lamina group, and (e) for the 1.0 mm lamina group; different letters above the median indicate statistically significant pairwise comparison (*p* < 0.05); pairwise comparisons were carried out using the Dunn–Sidak test, and the *p*‐value was corrected using the Bonferroni method. NS: Statistically not significant (*p* ≥ 0.05).

* Statistically significant (*p* < 0.05).

**Table 3:** Quantitative histomorphometric analysis for defect closure %.

Closure (%)	Group					Test of significance *p*‐value
	Control (a)	Single collagen (b)	Double collagen (c)	0.5 mm lamina (d)	1 mm Lamina (e)	
**No bone graft group**						
– *n*	10	10	8	10	10	*H*_(df=4)_ = 45.102
– Min–max	9.60–11.00	27.50–30.30	40.30–43.00	47.60–51.50	62.30–65.20	*p* < 0.001*
– Median	10.25^a,b^	28.85^a,b,c^	42.05^b,c,d^	49.90^c,d,e^	64.05^d,e^	
– 25th –75th percentile	9.90–10.70	28.00–29.50	41.05–42.35	48.90–50.40	63.20–64.50	
**Bone graft group**						
– *n*	9	9	10	8	6	*H*_(df=4)_ = 39.262
– Min–max	16.10–17.50	43.90–46.80	57.20–59.10	68.30–71.00	84.00–86.50	*p* < 0.001*
– Median	16.80^a,b^	45.10^a,b,c^	58.20^b,c,d,e^	69.70^c,d,e^	85.45^c,d,e^	
– 25th –75th percentile	16.40–17.10	44.60–46.20	57.80–58.90	68.80–70.25	84.70–86.10	
Test of significance	*Z*_(MW)_ = 3.674	*Z*_(MW)_ = 3.676	*Z*_(MW)_ = 3.554	*Z*_(MW)_ = 3.554	*Z*_(MW)_ = 3.254	
*p*‐value	*p* < 0.001*	*p* < 0.001*	*p* < 0.001*	*p* < 0.001*	*p* < 0.001*	

*Note: n*: Number of patients. Min–max: Minimum–maximum. *Z*_(MW)_ = *Z* of the Mann–Whitney *U* test. H = Kruskal‐Wallis H Test. Pairwise comparisons: Superscript alphabets (a, b, … etc.) are assigned to groups in sequence: (a) for the control group, (b) for the single collagen group and (c) for the double collagen group, (d) for the 0.5 mm lamina group, and (e) for the 1.0 mm lamina group; different letters above the median indicate statistically significant pairwise comparison (*p* < 0.05); pairwise comparisons were carried out using the Dunn–Sidak test, and the *p*‐value was corrected using the Bonferroni method. NS: Statistically not significant (*p* ≥ 0.05).

* Statistically significant (*p* < 0.05).

### 3.6 BG Group (*N* = 42)

Histologically, remnants of the membrane were present in the SC subgroup. Small fragments of bone graft associated with newly formed bone were observed centrally; however, the surgical area remained largely open, with minimal new bone formation at the periphery. A mild inflammatory infiltrate composed mainly of lymphocytes and macrophages, with scattered osteoclasts, was noted around the graft particles.

In the DC subgroup, membrane remnants were present, and newly formed bone appeared more mature and abundant than in SC. Bone trabeculae were organized in discrete islands separated by thin bundles of connective tissue, with partial continuity to adjacent pristine bone. No significant inflammation was observed.

In the L1 subgroup, less than 50% of the cortical lamina remained. Loose fibrous connective tissue occupied the surgical area, containing scattered regions of new bone associated with residual graft particles. No clear continuity between the newly formed bone and the adjacent pristine bone was observed.

In contrast, the L2 subgroup demonstrated extensive new bone surrounding the graft material and occupying the full defect area, with continuity to the adjacent native bone. Occasional chronic inflammatory infiltrates were noted around isolated graft particles. Residual lamina fragments were also present.

In the BG control group, the defect was largely filled with loose fibrocollagenous tissue, showing the least new bone among all subgroups. New bone was confined to the defect margins and around isolated graft fragments, without continuity to the native bone.

Histologic scores increased progressively across subgroups, with L2 exhibiting the highest values and the control the lowest. The Kruskal–Wallis test confirmed significant differences among the subgroups (*p* < 0.001).

### 3.7 Quantitative Histomorphometry

Bone fill and defect closure medians increased from the control subgroup through SC, DC, and L1, and were highest in L2: bone fill 89.5% (IQR 88.2%–90.3%) and defect closure 85.5% (IQR 84.7%–86.1%). Significant intergroup differences were identified using the Kruskal–Wallis test (*p* < 0.001). Mann–Whitney *U* tests confirmed that bone grafting significantly enhanced both parameters across experimental subgroups (*p* < 0.001). Detailed values are presented in Tables 3 and 4.

At 24 weeks, thin eosinophilic fragments of residual cortical lamina were occasionally visible in L1 and L2 specimens. These remnants were integrated within the regenerated bone and surrounded by osteoid seams. No evidence of outward bulging or augmentation beyond the native calvarial contour was observed.

Pairwise comparisons demonstrated significant differences between the control subgroup and DC, L1, and L2 in the BG group, and between SC and L2. Comparisons within each barrier category consistently showed superior values in the grafted subgroups. Complete pairwise results are presented in Table 4.

**Table 4:** Comparison of histological score percentages.

Score (%)	Group					Test of significance *p*‐value
	Control (a)	Single collagen (b)	Double collagen (c)	0.5 mm lamina (d)	1 mm lamina (e)	
**No bone graft group**						
– *n*	8	8	6	8	8	
– Min–max	7.14–14.29	28.57–42.86	42.86–50.00	57.14–64.29	78.57–85.71	*H*_(df=4)_ = 35.899
– Median	7.14^a,b,c^	32.14^a,b,c^	46.63^a,b,c,d,e^	57.14^c,d,e^	78.57^c,d,e^	*p* < 0.001*
– 25th –75th percentile	7.14–7.14	28.57–35.71	42.86–50.00	57.14–60.71	78.57–85.71	
**Bone graft group**						
– *n*	7	7	8	6	4	
– Min–max	7.14–14.29	35.71–42.86	50.00–57.14	57.14–78.57	85.71–100.00	*H*_(df=4)_ = 29.790
– Median	14.29^a,b,c^	42.86^a,b,c^	53.57^a,b,c,d,e^	71.43^c,d,e^	85.71^c,d,e^	*p* < 0.001*
– 25th –75th percentile	7.14–14.29	42.86–42.86	50.00–57.14	64.29–78.57	85.71–92.86	
Test of significance	*Z*_(MW)_ = 1.788	*Z*_(MW)_ = 2.801	*Z*_(MW)_ = 2.526	*Z*_(MW)_ = 2.500	*Z*_(MW)_ = 2.173	
*p*‐value	*p* = 0.077 NS	*p* = 0.005*	*p* = 0.012*	*p* = 0.020*	*p* = 0.030*	

*Note: n*: Number of patients. Min–max: Minimum–maximum. *Z*_(MW)_ = *Z* of the Mann–Whitney *U* test. H = Kruskal–Wallis H Test. Pairwise comparisons: Superscript alphabets (a, b, … etc.) are assigned to groups in sequence: (a) for the control group, (b) for the single collagen group and (c) for the double collagen group, (d) for the 0.6 mm lamina group, and (e) for the 1.0 mm lamina group; different letters above the median indicate statistically significant pairwise comparison (*p* < 0.05); pairwise comparisons were carried out using the Dunn–Sidak test, and the *p*‐value was corrected using the Bonferroni method. NS: Statistically not significant (*p* ≥ 0.05).

* Statistically significant (*p* < 0.05).

## 4 Discussion

This study utilized a rabbit calvarial critical‐size defect model, defined as the smallest intraosseous defect that does not heal spontaneously over the animal's lifetime without intervention (Schmitz and Hollinger 1986). In rabbits, defects measuring 8–10 mm in diameter and 1.7–2 mm in depth are widely accepted as non‐healing and therefore suitable for evaluating regenerative strategies (Kotagudda Ranganath et al. 2022; Spicer et al. 2012). The rabbit calvarial model is well established for GBR research due to its intramembranous bone origin, standardized defect geometry, and translational relevance to human craniofacial anatomy (Buss et al. 2023; Delgado‐Ruiz et al. 2015; Tournier et al. 2021).

Rabbits are commonly selected for small‐animal bone regeneration studies because of their manageable size, rapid bone turnover, and skeletal maturity reached by 6 months of age (Anesi et al. 2020). Nevertheless, their accelerated bone metabolism may overestimate regenerative potential compared with humans, underscoring the need for cautious interpretation of preclinical outcomes (Tournier et al. 2021; Ma and Shen 2012)

While the rabbit calvarium provides a reproducible, non‐load‐bearing environment ideal for membrane testing, its healing dynamics differ from load‐bearing models. Small animals such as mice, rats, and rabbits exhibit rapid metabolic turnover and faster intramembranous bone formation, whereas larger animals rely more heavily on medullary‐driven repair and require longer healing periods (Hirohata et al. 1989; Gao et al. 2023). Load‐bearing models (e.g., rat tibia) allow assessment under mechanical stress but are limited by smaller defect sizes and different biological behavior (Kim and Kim 2013). The calvarial defect model was therefore selected for this study because it allows consistent defect standardization and remains one of the most widely used platforms for preclinical evaluation of barrier membranes.

The comparison of grafted versus non‐grafted osseous defects is well established in preclinical bone regeneration research, as it allows assessment of the true contribution of barrier membranes independent of the osteogenic effect of graft materials (Ferraz et al. 2015; Ma et al. 2020). Numerous in vivo studies have adopted similar designs to determine whether grafting confers additional benefit in well‐contained critical‐size defects (Zhao et al. 2020, 2024). This comparative framework is particularly important when evaluating novel barrier materials, as it helps clarify whether the membrane alone can support bone formation or whether adjunctive grafting is necessary for optimal outcomes (Wang and Carroll 2001).

A xenogeneic cortical bone barrier was selected because of its documented osteoconductive properties, slow resorption profile, and extensive clinical application in GBR, making it a biologically and clinically relevant material for evaluating barrier performance (Cordaro et al. 2008; Jensen and Terheyden 2009; Naser et al. 2024). Barrier stabilization is fundamental in GBR: it preserves the blood clot, prevents micromovement, and ensures selective osteogenic cell migration while excluding fibroblasts, thereby minimizing fibrous tissue ingrowth. These principles, as outlined in the PASS framework Primary closure, Angiogenesis, Space maintenance, and Stability, are essential for predictable regenerative outcomes (Wang and Boyapati 2006).

Collagen membranes served as comparators due to their widespread clinical use, excellent biocompatibility, and predictable soft tissue integration (Barone et al. 2013). Their resorbable nature eliminates the need for a second surgery, but their relatively rapid degradation may limit their ability to maintain space in larger defects. To partially overcome this limitation, double‐layering techniques have been proposed to extend barrier function and improve structural stability, as demonstrated in both preclinical and clinical investigations (Barone et al. 2013; Jung et al. 2007; Zubery et al. 2007)

The significant differences observed between the NB and BG groups across all membrane‐protected subgroups but not in the controls highlight the fundamental role of the barrier membrane in GBR. Barrier membranes mechanically exclude undesirable soft tissue cells while preserving a stable, secluded compartment that favors osteogenic and angiogenic repopulation, thereby enabling true regeneration rather than fibrous repair (Buser et al. 1990; Kormas et al. 2022). This principle has been consistently demonstrated in human GBR studies showing predictable ridge augmentation when resorbable membranes are used in combination with xenogeneic grafts (Buser et al. 2011; Thoma et al. 2018). The present results corroborate this evidence, demonstrating that the membrane, along with the graft, drives the regenerative process through space maintenance and selective cellular repopulation. Furthermore, the superior performance of the cortical lamina barriers suggests that membrane architecture and thickness are important determinants of regenerative efficacy.

In the BG group, bone formation progressed more centrally than peripherally, reflecting the characteristic mechanism of creeping substitution in particulate grafts. The porous xenogeneic particles permitted early vascular infiltration and osteogenic activity within the graft core, allowing bone formation to occur not only from the margins but also throughout the defect. In contrast, spontaneous healing of oral intraosseous defects typically proceeds centripetally, with new bone forming from the existing bony walls, and the center mineralizes last (Sculean et al. 2019). This discrepancy is partly explained by anatomical context: calvarial augmentation occurs directly over the dura mater, a highly vascularized periosteal‐like tissue, whereas oral defects rely on more limited vascular access from the cortical walls and periosteum. Bone substitutes, therefore, play a compensatory role in oral GBR by providing an osteoconductive and angioconductive scaffold that helps overcome the restricted revascularization of central avascular zones. Consistent with Yip et al. (2015) and Kamal et al. (2019), the present findings complement rather than contradict clinical GBR dynamics, illustrating how grafting materials enhance both the speed and uniformity of bone regeneration by extending the intrinsic centripetal process into the central portion of the defect.

In this study, the findings demonstrated that after 6 months, both the NB and BG groups showed increased measurements of viable outcomes in tissue quality and maturity within the cortical bone barrier subgroups L1 and L2 compared to their respective control groups. Similarly, the SC and DC subgroups exhibited significantly improved histological scores compared to the control group, although the most favorable outcomes were observed in the cortical bone barrier subgroups, particularly L2.

The enhanced and favorable outcomes of the DC compared to the SC and control groups can be attributed to its prolonged barrier function and improved structural stability. Human and in vivo studies have confirmed that DC resists degradation, thus providing better space maintenance and minimizing soft tissue collapse, leading to enhanced regenerative results (Kim et al. 2009)

The superior tissue regeneration quality in the cortical bone barrier groups L1 and L2, compared to other groups, indicates that both the quantity and quality of bone formation were significantly enhanced by the use of cortical xenograft bone barriers.

The application of cortical barriers in bone regeneration offers several advantages, which are in accordance with previous studies (Rossi et al. 2017, 2023). One of the key benefits is their combination of rigidity and resilience, which facilitates dimensional preservation of the graft site and ensures optimal space‐making. This feature is critical during the phases of bone healing, as insufficient membrane sturdiness can lead to a reduction in the space available for new bone formation (Alvira‐González and De Stavola 2020; Lopez et al. 2018).

In this study, a progressive increase in bone regeneration outcomes was observed with increasing cortical barrier thickness. The L2 subgroup consistently demonstrated higher histologic scores, greater bone fill, and more complete defect closure compared to the SC and DC, L1 subgroups, indicating superior bone regeneration compared to thinner membranes made of both cortical and collagen materials (Deepika‐Penmetsa et al. 2017). These findings support the biological premise advocated by previous studies that thicker cortical lamina barriers provide superior space maintenance, mechanical stability, and resistance to early biodegradation, thereby creating more favorable osteoconductive and angiogenic conditions during healing (Nehal et al. 2024). While this study does not advocate the routine use of duplicate membranes, the bone regeneration associated with the 1‐mm cortical lamina suggests that thicker, more rigid barriers are particularly advantageous in clinical situations requiring substantial space maintenance where mechanical stability and prolonged barrier function are critical for predictable bone regeneration.

It is important to note that although fixation pins used with resorbable collagen and lamina membranes may occasionally require removal, these systems still demonstrate fewer complications than non‐resorbable PTFE membranes. While PTFE offers excellent space maintenance, it is associated with higher infection rates and fibrous tissue formation (Hindryckx et al. 2025). In contrast, resorbable barriers stabilized with pins provide predictable regeneration without the long‐term complications or re‐entry procedures often required for non‐resorbable membranes (Urban et al. 2025). This concludes that using xenogenic cortical lamina could be advantageous in situations that require rigid membranes, albeit Ti‐mesh or PTFE (Yu et al. 2023)

Previous studies have reported mixed outcomes regarding the effectiveness of bone grafting in well‐contained defects (Ferraz et al. 2015; Zhao et al. 2024). In the present study, however, both grafted and non‐grafted defects demonstrated enhanced regeneration when combined with cortical membranes, with the greatest effect observed in the thicker lamina. This underscores that the barrier itself, not only the graft, plays a decisive role in directing bone regeneration.

An important observation in this study was the higher complication rate in the BG group, particularly within the L2 subgroup. The increased incidence of dehiscence and infection likely reflects technical challenges associated with closing flaps over thicker, more rigid membranes. Similar concerns have been reported by Rossi et al. (2017) and Schuh et al. (2021), who emphasized the technique sensitivity of cortical laminae. These findings highlight the need for meticulous flap design and tension‐free closure when thicker barriers are used. Future studies should investigate how keratinized tissue width, flap design, and suturing techniques influence the risk of membrane exposure.

Despite these complications, statistical power was preserved due to planned oversampling, ensuring that subgroup comparisons remained valid.

The translation of these findings to clinical practice must consider several anatomical and surgical variables. In humans, tension‐free closure is more difficult to achieve in the oral cavity, and insufficient keratinized tissue may predispose to membrane exposure. The human oral microbiome, which is far more complex than the controlled environment in animal models, may also increase the risk of infection, especially when primary closure is compromised. These factors emphasize the importance of individualized treatment planning and careful soft‐tissue management when applying thicker membranes in GBR. Further investigation into models that better approximate oral conditions is warranted.

This study did not include a quantitative assessment of barrier resorption, as specimens were evaluated at a single 24‐week time point. Although residual fragments of the thicker lamina (L2) were occasionally visible histologically, they remained integrated within the regenerated bone and did not extend beyond the native bony envelope. Sequential‐time‐point designs and volumetric or histochemical analyses are recommended to describe the resorption kinetics of cortical xenograft barriers more precisely.

The semiquantitative histologic scoring system used in this study provides a comprehensive overview of regenerative quality by integrating several tissue parameters into a single index. However, future work should analyze each subcomponent individually to better characterize the specific biological contributions of different barrier materials.

## 5 Conclusions

This in vivo study assessed the regenerative potential of collagen and cortical xenogeneic barrier membranes of varying thicknesses in rabbit calvarial critical‐size defects over 6 months. The findings demonstrated that cortical bone lamina barriers with thicknesses of 0.5 mm and 1.0 mm were associated with improved tissue quality and maturity compared to single and double‐layered collagen membranes. Notably, these effects were observed even in the absence of bone grafting. These findings warrant further investigation to determine their applicability in clinical settings. Importantly, the mechanical and dimensional characteristics of barrier membranes, particularly their thickness, emerge as critical variables influencing the predictability and effectiveness of bone regeneration strategies.

## Author Contributions

Abdelsalam Elaskary contributed to the overall conception and design. Abdelsalam Elaskary, Rola Al Habashneh, Tasneem Sharairi, and Mohammed Alorjani were involved in the data acquisition and interpretation of data. Ahmed Zaki was responsible for animal caretaking, handling, and anesthesia. Abdelsalam Elaskary, Moataz Meabed, Mohammed Alorjani, and Tasneem Sharairi performed the surgical procedures. Malek Hudieb contributed to the surgical procedures. Tasneem Sharairi was involved with the histologic sectioning and staining. Mohammed Alorjani was responsible for histological data acquisition, photomicrographs, and histologic interpretation. Bassem Elfahl completed the quantitative measurements and data analysis. Mihad Ibrahim and Abdelsalam Elaskary led the writing. Abdelsalam Elaskary critically revised the manuscript. All authors gave final approval and agreed to be accountable for all aspects of the scientific work.

## Ethics Statement

All experimental procedures were approved by the Jordan University of Science and Technology Animal Care and Use Committee (JUST‐ACUC‐192) and the Institutional Animal Care and Use Committee, Alexandria Borg El‐Arab University Research Center (67‐4J‐9022), which conforms to the principles laid by the National Research Council Guide for the Care and Use of Laboratory Animals.

## Conflicts of Interest

The authors declare no conflicts of interest to report pertaining to this study except Elaskary, who is a lecturer for BioHorizons and Tecnoss Dental.

## Supporting information

Author_Checklist‐Full‐2.

## Data Availability

The data that support the findings of this study are available from the corresponding author upon reasonable request.
